# Bilateral ptosis as first presentation of cytophagic histiocytic panniculitis: a case report

**DOI:** 10.1186/s12886-017-0511-6

**Published:** 2017-07-01

**Authors:** Wang Liao, Songhua Xiao, Juanjuan Yong, Shengnuo Fan, Wenli Fang, Yuqiu Zheng, Jun Liu

**Affiliations:** 1 Department of Neurology, Sun Yat-sen Memorial Hospital, Sun Yat-sen University, Guangzhou, Guangdong 510120 China; 2 Department of Pathology, Sun Yat-sen Memorial Hospital, Sun Yat-sen University, Guangzhou, Guangdong 510120 China

**Keywords:** Cytophagic histiocytic panniculitis (CHP), Panniculitis-like T-cell lymphoma (SPTL), Lupus panniculitis, Ptosis, Chemotherapy, Case report

## Abstract

**Background:**

Cytophagic histiocytic panniculitis (CHP) is a rare form of nodular panniculitis that may progress to panniculitis-like T-cell lymphoma. We report a case of CHP that first manifested as bilateral ptosis, which is the first reported case of this presentation.

**Case presentation:**

A 25-year-old woman without medical history was referred to the neurology department of our hospital for evaluation of bilateral ptosis. Three months previously, she suddenly complained of bilateral ptosis without apparent cause. Simultaneously, non-painful tender subcutaneous nodules and eschar-like skin lesions were observed on her extremities and trunk. A diagnosis of CHP was made based on skin biopsy from the left thigh showing lobular panniculitis, vasculitis, and adiponecrosis, with infiltration of inflammatory cells, including lymphocytes, histiocytes, and phagocytic histiocytes. Her condition continued to worsen with corticosteroid and immunosuppressive agent (thalidomide) treatment. Significant improvement was noticed after three cycles of chemotherapy of THP-COP (pirarubicin, cyclophosphamide, vincristine, and prednisolone).

**Conclusions:**

CHP is a rare condition whose clinical presentation may include bilateral ptosis and biopsy is required for diagnosis of CHP.

## Background

Cytophagic histiocytic panniculitis (CHP) is a rare form of nodular panniculitis, histologically characterized by subcutaneous adipose tissue infiltration by benign-appearing cytophagic macrophages called “bean bag” cells. These cytophagic macrophages are known to engulf blood cells, including lymphocytes, erythrocytes, and platelets [1]. Various clinical manifestations have been described for CHP: subcutaneous nodules, fever, hepatomegaly, splenomegaly, lymphadenectasis, leukopenia, thrombocytopenia, anemia, elevated liver enzymes, and hemorrhagic diathesis [2]. There are less than 100 reported cases of CHP which is frequently fatal although histologically benign [3]. According to the analysis of 37 CHP patients by Ito, the mortality rate of CHP was 43.2%. However, the death of patients before 1989 (60.0%) was greater than after 1990 (23.5%), which could be attributed to the difference of treatment strategy [2]. Accurate diagnosis is therefore crucial to improve patient prognosis. Herein we report a case of biopsy-proven CHP that first manifested as bilateral ptosis, which had never been reported previously.

## Case presentation

**Fig. 1:**
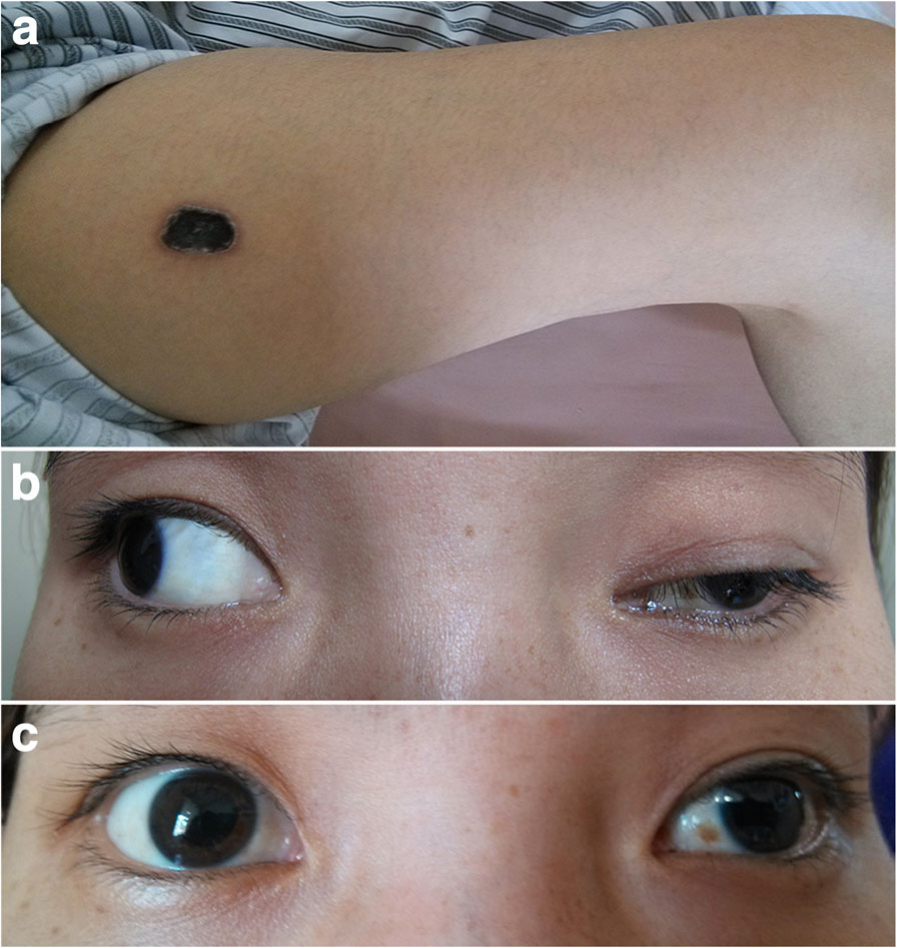
Physical examination. **a** Large ulcerated lesions and eschar on the anterior thigh. **b** Ptosis and eyes position of the patient before treatment; and **c**. After treatment

**Fig. 2:**
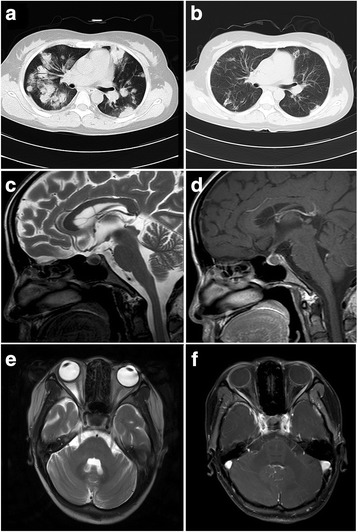
Magnetic resonance imaging (MRI) data. **a** Chest computed tomography before treatment; **b** Chest computed tomography after treatment. **c** Round signal (8 × 6 mm) with a clear edge on the posterior pituitary. **d** Mild dynamic contrast-enhancement in the delayed phase. **e** Axial T2 MRI showing the optic nerve cut. **f** T1, postcontrast MRI with fat suppression showing the optic nerve cut

A 25-year-old woman with no medical history presented to the neurological department of our hospital for evaluation of bilateral ptosis. Before that, she had visited several ophthalmologists, but no clear diagnosis was established. Three months previously, she suddenly complained of bilateral ptosis without any apparent cause. Simultaneously, non-painful tender subcutaneous nodules and eschar-like skin lesions were observed on her extremities and trunk with diameters of 5–15 mm (Fig. 1a). Neurological examination revealed bilateral ptosis and fixed mydriasis: left and right palpebral fissures were 7 and 12 mm, respectively. Both pupil diameters were 8 mm with no anisocoria. She also had ophthalmoplegia with left gaze palsy, upward and downward paresis of the right eye. Paresis of adduction of the lateral gaze in the left eye was observed (Fig. 1b). Laboratory data showed mild anemia, low growth hormones (insulin-like growth factor 1, factor binding protein 3), and low sex hormone levels (luteinizing hormone, estradiol, progesterone, testosterone), and significantly elevated levels of liver enzymes (alanine aminotransferase, aspartate aminotransferase, gamma-glutamyl transpeptidase). Autoimmune indicators including antinuclear antibody, antibody to double-stranded DNA, and complement level were normal. No remarkable findings were obtained after tuberculosis tests (T-spot) and cerebrospinal fluid (CSF) examination. Hepatitis virus, human immunodeficiency virus, syphilis, and rubella virus serology tests were negative, but the patient was positive for cytomegalovirus, herpes simplex virus, and Epstein-Barr (EBV) IgG antibodies. There was no hepatosplenomegaly or lymphadenopathy as assessed by ultrasound. Magnetic resonance imaging showed an abnormal signal in the pituitary gland without significant change for optic nerve (Fig. 2). Retinal examination was normal. The right and left eyes had visual acuities of 5/10 and 7/10, respectively.

**Fig. 3:**
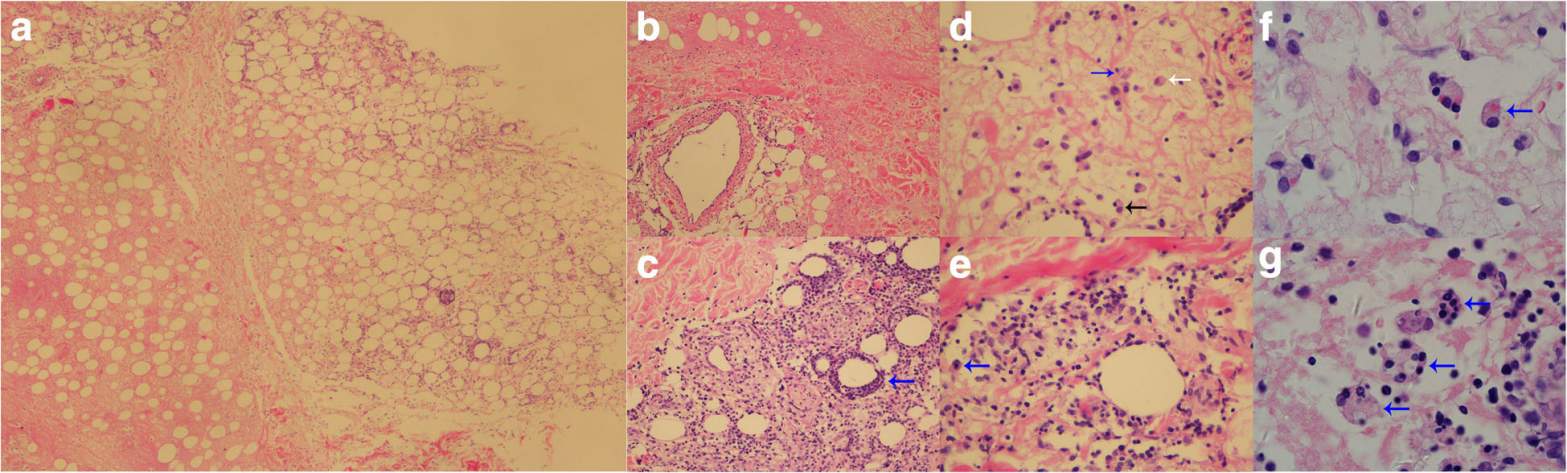
Histology findings. **a** Overall view of the biopsy section (hematoxylin and eosin [H&E] stain × 40). **b** Fat necrosis (H&E stain × 100). **c** Perivasculitis with infiltration of lymphocytes, neutrophils, plasma cell, histiocytes (*blue arrow*, H&E stain × 200). **d** Histiocytes phagocytizing erythrocytes (*white arrow*), leukocytes (*black arrow*), and karyorrhexis (*blue arrow*) (H&E stain × 400). **e** Phagocytic histiocytes containing leukocytes (*blue arrow*, H&E stain × 400). **f** Higher magnification of erythrophagocytic histiocytes within adipose tissue (*blue arrow*, H&E stain × 1000). **g** “Bean bag” cells phagocytosing leukocytes, lymphocytes, adipose tissue, nuclear debris (*blue arrow*, H&E stain × 1000)

A skin biopsy from the left thigh showed a substantial lobular panniculitis and perivasculitis (mainly dermal veins involved) with infiltration of lymphocytes, neutrophils, plasma cells and histiocytes. There were numerous infiltrating lymphocytes, plasma cells, eosinophils and macrophages in the subdermal fat tissue. The phagocytic histiocytes (“bean bag” cells) phagocytosing erythrocytes, leukocytes, platelets and nuclear debris showed no atypical cytology. Both liquefactive and coagulative local adiponecrosis were observed. Some adipocytes were rimmed by lymphocytes in a ring-like structure (Fig. 3). Immunohistochemical examination showed results of CD4, CD8, CD 56 (a marker of natural killer T cells) and monoclonal rearrangement of the T-cell receptor were negative while that of Epstein-Barr virus (EBV) EBER-1 positive. Examination of CD123 was not performed [4]. Aspiration of bone marrow was normal. Therefore, a diagnosis of CHP was made.

Intravenous corticosteroid therapy was administered for 7 days, followed by oral corticosteroid treatment. However, no improvement was obtained; instead, she developed fever with progressively worsening pulmonary infiltration. Her condition continued to worsen, even with the addition of an immunosuppressive agent (thalidomide). Therefore, the treatment was switched to chemotherapy. Though CHOP (cyclophosphamide, doxorubicin, vincristine, and prednisolone) [5] is the most common chemotherapy regimen, we decided to use THP-COP (pirarubicin, cyclophosphamide, vincristine, and prednisolone) for its proven superior efficacy. Pirarubicin (THP) is an analogue of doxorubicin [5]. Significant improvement was noticed after three cycles of chemotherapy, with partially recovery of ptosis, eye movements, and decreased pupil diameters (Fig. 1c). A decrease in the number of subcutaneous nodules and recovery of hepatic function and lung performance were obtained and no relapse has happened through June 2015 (Fig. 2b). However, her visual acuity has remained the same.

## Discussion

We described a case of biopsy-proven CHP with a first presentation of bilateral ptosis, which is the first reported case of this condition. Like the patients in previous cases, this patient had subcutaneous nodules. The decline in her hormone levels, which might be caused by a pituitary injury, is also rare.

The etiology of CHP remains unclear. It might be triggered in response to an unknown T cell disorder [6]. Recently, CHP was suggested to be a cutaneous manifestation of hemophagocytic syndrome or a natural disease progression of SPTL likely associated with EBV infection [7, 8]. The pathological examination of subcutaneous nodules in this case showed no atypical lymphocytes, indicating that a diagnosis of SPTL was insufficient. In addition, immunohistochemical examination showed that specific immuno-phenotypic markers of NK/T-cell lymphoma, which can present itself as SPTL, were not present. Considering the remission of the patient, we assume that the dysfunction of the second and third cranial nerves was caused by an inflammatory process or CHP-induced compression because we could not determine any other causative factor. A biopsy specimen from the central nerve system is the best way to confirm that; however, we were unable to obtain one without consent from the patient.

The differential diagnoses include infection (especially tuberculosis), tumor, histiocytosis, neurosarcoidosis and lupus panniculitis [8, 9]. A skin biopsy is therefore necessary to make the diagnosis. Systemic lupus erythematosus (SLE) is an autoimmune process which can affect the eye and visual system in 20% of individuals. Both panniculitis and ptosis are rare clinical entities in the SLE spectrum. However, this patient had no medical history of SLE and got no response to corticosteroid treatment. In addition, results of autoimmune indicators including antinuclear antibody, antibody to double-stranded DNA, and complement level were normal. Most importantly, the histological findings were different from those of lupus panniculitis: characteristic phagocytic histiocytes (“bean bag” cells) and coagulative necrosis were showed without focal calcifications or cysts bounded by amorphic eosinophilic material. Thus, lupus panniculitis could be excluded [10–12].

Until now, very few studies have been conducted can guide the management of this disease. Some patients recover after treatment with a combination of prednisone, cyclosporine, and chemotherapy [9, 13, 14]. In refractory cases, the efficacy of other immunosuppressive therapeutics [14], plasmapheresis [4], interleukin-1 (IL-1) receptor antagonists [15], and autologous peripheral blood stem cell transplantation has been reported [9]. The patient reported here had remission after treatment with THP-COP.

## Conclusions

CHP is a rare condition whose clinical presentation may include bilateral ptosis. Biopsy is required for diagnosis of CHP.

## Abbreviations

CHOP: cyclophosphamide doxorubicin vincristine and prednisolone
CHP: Cytophagic histiocytic panniculitis
CSF: cerebrospinal fluid
EBV: Epstein-Barr
SPTL: panniculitis-like T-cell lymphoma
THP: Pirarubicin
